# Influence of nutrients and metabolites on the differentiation of plasma cells and implications for autoimmunity

**DOI:** 10.3389/fimmu.2022.1004644

**Published:** 2022-11-18

**Authors:** Bandik Föh, Jana Sophia Buhre, Christian Sina, Marc Ehlers

**Affiliations:** ^1^ Department of Medicine I, University Hospital Schleswig-Holstein, Lübeck, Germany; ^2^ Institute of Nutritional Medicine, University of Lübeck and University Hospital Schleswig-Holstein, Lübeck, Germany; ^3^ Airway Research Center North, University of Lübeck, German Center for Lung Research Deutsches Zentrum für Lungenforschung (DZL), Lübeck, Germany

**Keywords:** plasma cells, nutrients, metabolites, autoimmunity, antibodies, IL-10, IgG glycosylation, metabolism

## Abstract

The modulation of inflammatory (auto)immune reactions by nutrients and gut bacterial metabolites is of great interest for potential preventive and therapeutic strategies. B cell-derived plasma cells are major players in inflammatory (auto)immune responses and can exhibit pro- or anti-inflammatory effects through (auto)antibody-dependent and -independent functions. Emerging evidence indicates a key role of nutrients and microbial metabolites in regulating the differentiation of plasma cells as well as their differentiation to pro- or anti-inflammatory phenotypes. These effects might be mediated indirectly by influencing other immune cells or directly through B cell-intrinsic mechanisms. Here, we provide an overview of nutrients and metabolites that influence B cell-intrinsic signaling pathways regulating B cell activation, plasma cell differentiation, and effector functions. Furthermore, we outline important inflammatory plasma cell phenotypes whose differentiation could be targeted by nutrients and microbial metabolites. Finally, we discuss possible implications for inflammatory (auto)immune conditions.

## Introduction

Inflammatory autoimmune diseases are a significant burden to individual patients and health care systems worldwide. They are frequently characterized by the appearance of antibodies (Abs) directed against self-antigens that are generated by B cell-derived plasma cells (PCs) (1). Inflammatory autoimmune diseases include, but are not limited to (seropositive) rheumatoid arthritis (RA), systemic lupus erythematosus (SLE), diabetes mellitus type 1, polymyositis, autoimmune hepatitis, and multiple sclerosis (MS) (2). Furthermore, for other inflammatory diseases, such as inflammatory bowel disease (IBD), specific autoAbs have not yet been identified, but inflammatory T and B cell responses, as well as Abs directed against gut microbes or pharmaceuticals, are implicated in their pathophysiology (3, 4).

PCs can exert pro- or anti-inflammatory effects *via* Ab-dependent and -independent effects, including the secretion of (anti-)inflammatory cytokines (5, 6). Thus, strategies for inhibiting or shifting (auto)antigen-specific inflammatory T and B cell responses and the resulting PCs to a less or even anti-inflammatory phenotype are of high relevance.

Since activated B cells and PCs rely heavily on high energy turnover (7), especially in comparison to naïve B cells (8, 9), B cell-activating signals lead to increased uptake of nutrients (7). Thus, nutrients are canonically considered to play a passive, but important role as substrates and/or building materials for PC metabolism and function (7). In addition, the influence of nutrients and gut bacterial metabolites on B cell activation and PC differentiation as well as differentiation to pro- versus anti-inflammatory PC subtypes are becoming increasingly evident. Nutrients and metabolites might thereby act indirectly on different immune cells or directly on B cells to influence B cell activation and PC differentiation.

Here, we summarize known influences of nutrients and microbial metabolites on B cell-intrinsic signaling pathways of B cell activation and differentiation to PCs and their pro- or anti-inflammatory phenotypes. In addition, we present important inflammatory PC phenotypes whose differentiation might be targeted by nutrients and microbial metabolites. Finally, we discuss possible implications for inflammatory autoimmune disorders.

## B cell activation and plasma cell differentiation

B cell activation and PC differentiation from naïve B cells are tightly controlled *via* a complex network of intracellular transcriptional regulators (10). PC differentiation can occur extrafollicularly or *via* an intermediate stage in the germinal center (GC) reaction, where class switching and in particular affinity maturation occur. The intermediate stage in the GC is characterized, e.g., by high expression of the transcription factors B cell lymphoma 6 protein (BCL6) (11, 12), interferon regulatory factor 8 (IRF8) (13, 14), and activation-induced cytosine deaminase (AID) (15), whereas PC differentiation in general is characterized, e.g., by the expression of the transcription factors interferon regulatory factor 4 (IRF4) (16, 17) and B lymphocyte-induced maturation protein-1 (BLIMP-1) (18–20). Both extrafollicular and intrafollicular PC differentiation are influenced by metabolic signaling pathways.

**Figure 1:**
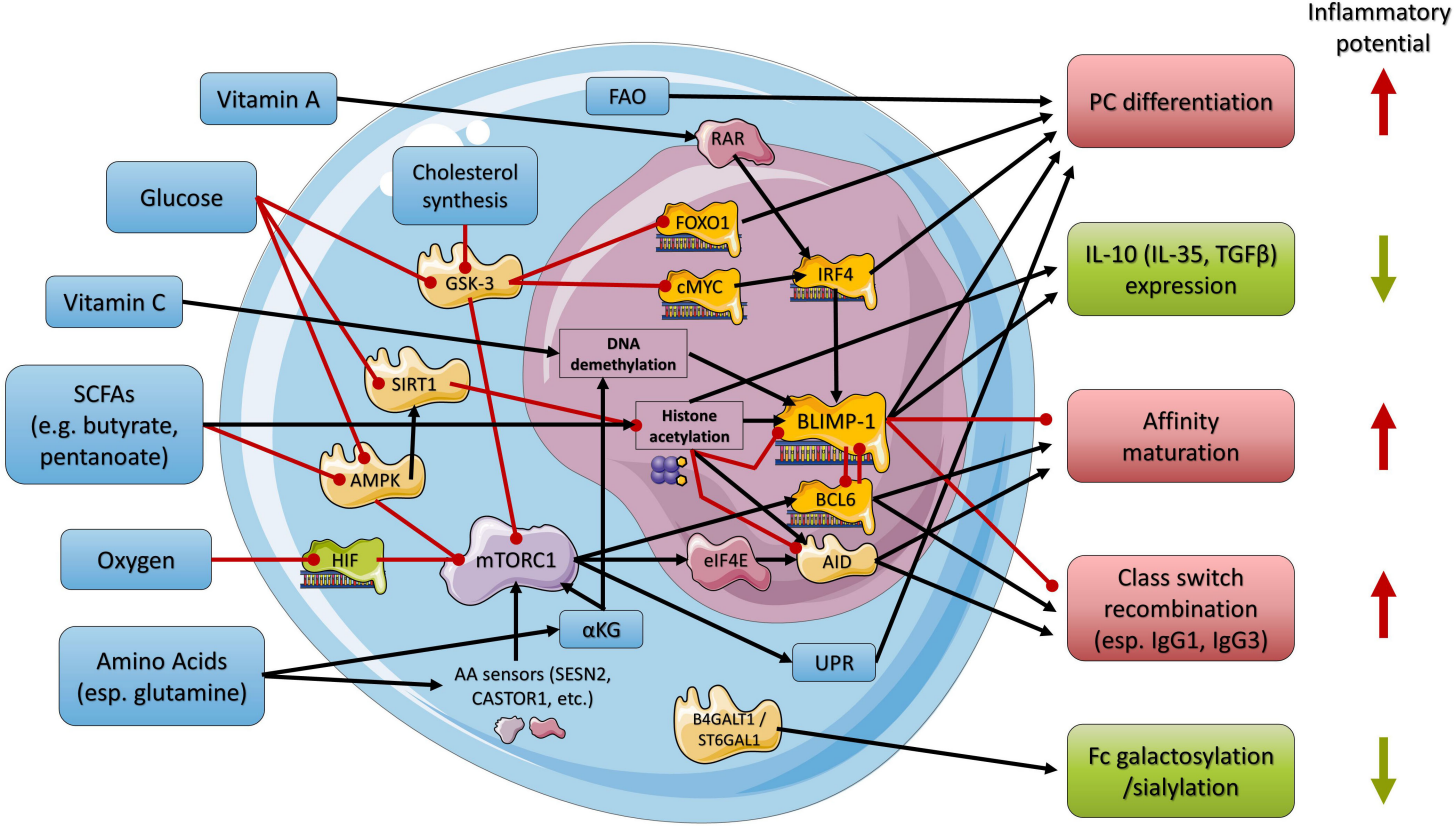
Simplified overview of the regulatory network used by nutrients and metabolites in regulating B cell maturation and PC differentiation and function. Black arrows indicate stimulation, and red arrows indicate inhibition. Nutrients, metabolites, and related cellular processes are indicated in blue, enzymes in light yellow, transcriptional regulators in orange, and other proteins in shades of purple. Anti- or proinflammatory outcomes of regulatory processes are indicated in green or red, respectively. AID, activation-induced cytidine deaminase; αKG, α-ketoglutarate; AMPK, AMP-activated protein kinase; B4GALT1, beta-1,4-galactosyltransferase 1; BCL6, B-cell lymphoma 6; BLIMP-1, B lymphocyte-induced maturation protein-1; cMyc, MYC proto-oncogene; eIF4E, eukaryotic translation initiation factor 4E; FAO, fatty acid oxidation; FOXO1, Forkhead-Box-Protein; O1GSK-3, glycogen synthase kinase 3; HIF, hypoxia-inducible factor; IRF4, interferon regulatory factor 4; mTORC1, mammalian (mechanistic) target of rapamycin complex 1; RAR, retinoic acid receptor; SIRT1, sirtuin 1; ST6GAL1, beta-galactoside alpha-2,6-sialyltransferase 1; UPR, unfolded protein response.

Glucose is a crucial energy substrate during lymphopoiesis, inducing B cell development and function while reducing apoptosis (21). Its uptake and metabolization are rapidly upregulated after B cell activation (9, 22, 23). Glucose inhibits AMP-activated protein kinase (AMPK), a metabolic sensor that is activated by glucose restriction (24) (**Figure 1**). In its activated form AMPK inhibits several downstream targets, including mammalian (mechanistic) target of rapamycin complex 1 (mTORC1) (25). mTORC1, in turn, induces BCL6, which is crucially important for B cell maturation prior to PC differentiation (15, 26). Moreover, mTORC1 contributes to the immediate unfolded protein response (UPR), preparing B cells for PC differentiation and Ab secretion (27). Accordingly, deletion of the AMPKalpha1 subunit increases the primary Ab response, while disruption of mTORC1 impairs PC differentiation from activated B cells and Ab responses (15, 28, 29).

Similarly, glucose inhibits glycogen synthase kinase 3 (GSK-3), another metabolic sensor for glucose availability, which is also activated in glucose-restricted conditions (**Figure 1**). In its activated form GSK-3 represses forkhead box protein O1 (FOXO1) and MYC proto-oncogene (c-MYC), thereby limiting the expression of key transcription factors of PC differentiation including IRF4 and favoring B cell quiescence (28–30). Consequently, glucose restriction might inhibit B cell activation and PC differentiation.

Amino acids serve as additional essential energy substrates (esp. glutamine) but also as building blocks for the Ab-producing machinery (7). Similar to glucose, amino acid uptake (esp. glutamine and leucine) and metabolization are highly upregulated in activated B cells and PCs (7, 31). Glutamine was reported to be essential for the differentiation of PCs *in vitro* as early as 1985 (32). Furthermore, the amino acids glutamine, arginine, leucine, and methionine elicit a complex signaling cascade involving several cytosolic and lysosomal amino acid (AA) sensors (e.g., Sestrin-2 (SESN2); Leucyl-tRNA Synthetase (LRS); Cytosolic arginine sensor for mTORC1 subunit 1 (CASTOR1); and S-adenosylmethionine sensor upstream of mTORC1 (SAMTOR)) that converge on Rag GTPases and activate mTORC1 (33–35) (**Figure 1**). Additionally, glutamine metabolism, as well as ATP production *via* the citric acid cycle, generates α-ketoglutarate (αKG) (36). αKG acts as a cofactor in demethylation processes at histone H3K27me3, thereby activating the transcription of the *BCL6* gene (37). Moreover, αKG might activate mTORC1 (36, 38). Together, these pathways of amino acid metabolism might contribute to B cell activation and PC differentiation. In contrast, protein-energy-malnutrition might lead to changed/reduced PC responses.

In addition, oxygen availability is a key factor for PC differentiation. Sustained hypoxia or experimental stabilization of hypoxia-inducible factor (HIF) inhibits mTORC1 thereby reducing the proliferation and survival of activated B cells (31) (**Figure 1**).

Thus, mTORC1 serves as a (positive) metabolic sensor potentially integrating glucose, amino acid, and/or oxygen availability to prepare B cells for PC differentiation (15, 26). Notably, the function of mTORC1 in PC differentiation is further dependent on adequate mitochondrial function, particularly oxidative phosphorylation (39).

Another significant source of energy for B cells prior to PC differentiation is fatty acid oxidation (FAO) (40), making the availability of corresponding substrates a possible metabolic checkpoint in regulating PC differentiation (**Figure 1**). Indeed, pharmacological inhibition of FAO decreased the B cell population with intermediate mitochondrial mass and membrane potential, which is predetermined for PC differentiation, and consequently also reduced the differentiation of PCs *in vitro* (41).

Furthermore, vitamin A and its metabolite all-trans retinoic acid have diverse effects on B cell development, PC differentiation, and the Ab response (42, 43), including PC differentiation and Ab production *via* activation of the retinoic acid receptor (RAR) and subsequent induction of IRF4 (44, 45) (**Figure 1**).

Another essential nutrient, ascorbic acid (vitamin C), promotes PC differentiation and Ab responses *via* an epigenetic pathway involving increased DNA demethylation by Tet methylcytosine dioxygenase 2 and 3 (TET2/3), leading to increased BLIMP-1 expression (46) (**Figure 1**). Moreover, it was suggested that the presence of vitamin C during early B cell activation predisposes GC B cells toward the PC lineage (46).

In summary, the availability of several nutrients and metabolites is necessary for activation of different signaling pathways for B cell activation and PC differentiation. Potentially, a reduction of these factors might be a strategy to reduce the differentiation of PCs in inflammatory autoimmunity diseases.

## Antibody-independent plasma cell functions

In recent years, it has become evident that PCs have an immunomodulatory role also independent of Ab secretion (5, 6). PCs can influence inflammatory immune responses by secreting pro-inflammatory cytokines, including IL-17, or anti-inflammatory cytokines, including IL-10, IL-35, and TGFβ (5, 6). Although metabolic factors are involved in regulating B cell activation, PC differentiation, and Ab secretion, the metabolic regulation of Ab-independent functions remains less explored. Recent studies have started to shed light on metabolic pathways underlying the expression and secretion of the key anti-inflammatory cytokine IL-10 in B cells and PCs and therefore on the induction of IL-10^+^ regulatory B cells and PCs.

In one study, intracellular cholesterol synthesis (the multistep conversion of HMG-CoA to cholesterol) was reported to upregulate IL-10 expression in B cells and PCs (47). Specifically, geranylgeranyltransferase (GGTase) and its substrate geranylgeranyl pyrophosphate (GGPP), which is derived from the cholesterol synthesis pathway, promoted protein kinase B (AKT) activation, which in turn inhibited GSK-3 (47) (**Figure 1**). Inhibition of GSK-3 led, as described above for glucose, to activation of BLIMP-1 (47). These authors further showed that BLIMP-1 contributes to the induction of IL-10 (47). However, the data suggest that - in addition to BLIMP-1 - further discriminating signaling cascades favoring the induction of IL-10 might be involved but remain to be determined.

Short-chain fatty acids (SCFAs), which are gut microbial metabolites of complex carbohydrates (dietary fibers), have also been reported to induce anti-inflammatory IL-10^+^ B cells as well as IL-10^+^ PCs. In one study, the SCFA butyrate (C4) increased the mRNA expression of *Irf4* and *Prdm1*, the gene coding for BLIMP-1, and the anti-inflammatory cytokines *Il-10*, *Tgfb*, and *Ebi3*, the gene product of which is part of the anti-inflammatory cytokine IL-35, at the same time and induced IL-10^+^ PCs (48). In this study, PC differentiation as well as *Il-10, Tgfb*, and *Ebi3* transcription by butyrate were linked to the inhibition of the class I histone deacetylase 3 (HDAC3) activity and increased acetylation of histone H3 at lysine residue 27 (H3K27) (48) (**Figure 1**). Notably, the regulatory IL-10^+^ PCs induced by butyrate were mostly of the IgM type (48). The potential of propionate to induce anti-inflammatory cytokines were low in this study (48). Furthermore, butyrate-induced IL-10^+^ B cells were also linked to the reduction of B cell-dependent inflammatory autoimmune conditions (49).

Similarly, the SCFA pentanoate (C5) induced IL-10 in B cells and effectively suppressed inflammatory conditions in murine models of MS and colitis (50). Mechanistically, pentanoate increased mTOR expression but also enhanced histone acetyltransferase (HAT) activity (**Figure 1**).

Together, the specificity of SCFAs for different HDACs or HATs seems to be a crucial factor in the induction of anti-inflammatory IL-10^+^ B cells and PCs.

## Antibody-dependent plasma cell functions

As naïve B cells express IgM-type B cell receptors, the earliest PCs of an induced immune response generate IgM Abs. Subsequently, activated B cells in extrafollicular (T cell-independent and -dependent) or GC (T cell-dependent) immune reactions can undergo class switch recombination into other isotypes and subclasses: IgD, IgG1-4, IgA1-2, and IgE in humans and IgD, IgG1, IgG2a (or IgG2c), IgG2b, IgG3, IgA, and IgE in mice (51).

IgG (auto)Abs are frequently associated with inflammatory (auto)immune conditions. Accordingly, human IgG1 and IgG3 and murine IgG2a/c and IgG2b have strong activating potentials because of their high affinity and specificity to certain activating Fc receptors for IgG (FcγRs) and the complement C1q molecule (52–55). In contrast, human IgG4 and murine IgG1 show no interaction with C1q, have a higher affinity for the inhibitory FcγRIIB, and might inhibit hexamer formation of the other IgG subclasses, which seems to be a prerequisite for their aforementioned activation potentials (52–55).

Thus, (i) inhibition of (IgG) class switch recombination in general, (ii) inhibition of the switch to the activating human IgG1 and IgG3 (murine IgG2a/c and IgG2b) subclasses, or (iii) induction of human IgG4 (murine IgG1) might be potential targets to reduce inflammatory (auto)immune conditions by nutrients and gut microbial metabolites.

Paralleling its crucial role in preparing B cells for PC differentiation, mTORC1 is also essential for the expression of AID (15) (**Figure 1**), the key enzyme for class-switch recombination (CSR), as well as somatic hypermutation (SHM) (56), thereby promoting isotype switching and affinity maturation (15). Since the nutrient-sensing mechanisms for glucose, amino acids, and oxygen involve mTORC1 as described above, it seems likely that these metabolic factors are also involved in regulating CSR and affinity maturation.

Indeed, immunoglobulin class switching to murine IgG was dramatically decreased under glucose restriction *in vitro* (9). Similarly, glutamine restriction dramatically reduced CSR to murine IgG in an mTORC1-dependent manner (9, 57, 58). Downstream of mTORC1, these effects were mediated by eukaryotic translation initiation factor 4E (eIF4E) and eIF4E-binding proteins (4E-BPs), which promote the translation of AID from its mRNA transcripts (58) (**Figure 1**).

The mTORC1 pathway is additionally utilized by the oxygen response system to regulate CSR and affinity maturation (31). In this study, oxygen restriction diminished isotype switching and reduced the level of murine IgG by limiting the expression of AID, while simultaneously reducing the level of high-affinity Abs (31).

Furthermore, glucose can block the class III HDAC sirtuin 1 (SIRT1), leading to enhanced histone acetylation and higher AID expression (59). In glycolysis, nicotinamide adenine dinucleotide (NAD^+^), which is a crucial substrate for SIRT1 activity (60), is reduced to NADH. Thus, intracellular glucose metabolism leads to decreased NAD^+^ levels, reduced SIRT1 activity, and increased expression of AID, thereby promoting class switch recombination and somatic hypermutation (59, 60) (**Figure 1**).

As mentioned above, specific SCFAs such as butyrate can induce non-class-switched anti-inflammatory IL-10^+^IgM^+^ PCs. However, the influence of SCFAs on the induction of class-switched PC is controversial, which will be described in the following.

One study found that SCFAs can increase FAO in metabolic tissues (61) and another study described that SCFAs can reduce AMPK activity, thereby activating mTORC1 and inducing class-switched PCs (62) (**Figure 1**).

In contrast, other studies reported an upregulation of B cell-intrinsic microRNAs (miRNA) by the SCFAs butyrate and propionate *via* HDAC inhibition that blocked BLIMP-1 and AID expression, resulting in reduced class-switched PCs and (auto)Ab responses (63–65) (**Figure 1**). Notably, one study described different outcomes with different doses of the SCFAs. At low doses propionate and butyrate enhanced class-switching, while decreasing at higher doses over a broad physiological range, AID and Blimp1 expression, class-switching, somatic hypermutation and plasma cell differentiation responses (63).

The study that described the induction of IL-10^+^IgM^+^ PCs by butyrate in mice also showed in the same experiment that butyrate reduced the gene expression of *Aicda*, the gene product of which is AID, most likely also by inhibiting HDAC (3) activity (48). Interestingly, the switch to IgG2b Abs (and in tendency toward IgG2a/c) was reduced, whereas the level of IgG1 Abs seemed to be unchanged, suggesting that in particular the switch to activating IgG subclasses was diminished (48).

These findings suggest that the SCFA butyrate can enhance IL-10^+^IgM^+^ PC differentiation and simultaneously block class switching toward PCs expressing activating IgG subclasses, highlighting butyrate as a potential candidate to reduce pro-inflammatory and enhance anti-inflammatory PC responses.

However, further studies have to clarify the opposite findings on the induction of class-switched PCs by SCFAs. Possibly, different *in vivo* and *in vitro* settings during the investigation of SCFAs induced non-inflammatory (including IL-10^+^IgM^+^ PCs) versus inflammatory immune responses leading to opposite findings. In this context, the possibility that pro- and anti-inflammatory PC subtypes may derive from different B cell precursors (B2 versus B1 and marginal zone B cells) should be evaluated. Furthermore, the effects of different SCFA dosages need to be considered and verified as well (63).

Importantly, the specificity of SCFAs for different HDACs or HATs seems to be a crucial factor in determining the potential outcomes. Even more so, different HDACs seem to have opposite roles, since glucose-mediated inhibition of the HDAC SIRT1 enhanced AID expression, whereas SCFA-mediated inhibition of HDACs reduced AID expression by the upregulation of B cell intrinsic miRNAs (63–65).

The inflammatory potential of Abs is further crucially dependent on the type of glycans attached to their Fc region. Complex biantennary *N*-glycans consist of a core of *N*-acetylglucosamines and mannoses that can be modified by core fucose, a bisecting *N*-acetylglucosamine, and two terminal galactose residues that can be further capped by sialic acid. The current consensus is that Ab Fc *N*-galactosylation plus terminal sialylation dampens the inflammatory potential of IgG, IgA, and IgM Abs (66–70).

Accordingly, low galactosylation and sialylation levels of autoantigen-specific, as well as total IgG Abs, are linked to pro-inflammatory effector functions in autoimmune conditions, e.g., rheumatoid arthritis (RA), whereas galactosylated plus sialylated IgG Abs are associated with less inflammatory or even protective functions (71).

Mechanistically, terminally sialylated glycan chains at Asn 297 in the Fc region of IgG Abs reduce the affinity to activating FcγRs and increase the interaction with glycan-binding receptors of the C-type lectin receptor family resulting in inhibitory signals (59, 68). Fc sialylation of total blood IgG increases its immunological buffer potential to up-regulate the IgG inhibitory receptor FcγRIIb on immune cells, which decreases inflammatory effector functions of (auto)antigen-specific IgG (68, 72, 73). Accordingly, the therapeutic efficacy of intravenous immunoglobulins (IVIg; high amounts of pooled serum IgG from healthy donors to treat inflammatory (auto)immune conditions) has been attributed to the terminally sialylated IgG subfraction for re-establishing a non-inflammatory total IgG Fc pattern (74, 75).

Galactosylation and sialylation of Abs in PCs are exerted by specific enzymes (beta1,4-galactosyltransferase, B4GALT1; beta-galactoside alpha-2,6-sialyltransferase 1, ST6GAL1) (72, 73, 76–80) (**Figure 1**). Recent studies have shown that IgG Abs with low galactosylation and sialylation levels can be determined in GC reactions under the influence of IFNγ-producing T follicular helper (T_FH1_) cells and IL-17-producing T_FH17_ cells (66).

Thus, identifying nutrients, metabolites and metabolic pathways that increase autoantigen-specific and/or total Ab galactosylation and sialylation might be a potential strategy for reducing or re-directing inflammatory (auto)immune conditions.

In addition, the availability of the respective substrates might also significantly impact the Ab glycosylation patterns, and thus the inflammatory potential of Abs. For mannose residues it has been shown that PCs primarily use glucose as a substrate (81), indicating that glucose availability is already needed for the *N*-core glycosylation of Abs. Recently, supplementation with the sialic acid precursor N-acetyl-D-mannosamine (ManNAc) increased total blood IgG sialylation levels in mice (82). However, data on associations of anti-inflammatory Ab Fc galactosylation and sialylation with substrate availability currently remain sparse.

Notably, reports on associations of dyslipidemia with the risk of cardiovascular disease and atherosclerosis with decreased Ab sialylation patterns indicate a link between metabolic contributions and Ab Fc glycosylation patterns (83–86). Moreover, obesity versus fasting has been linked to the induction of inflammatory conditions and the reduction of IgG galactosylation and sialylation levels (87, 88). In addition, a high-fat diet (HFD) recently reduced total blood IgG sialylation levels (82), and a Western diet - rich in carbohydrates and fat – was linked to inflammatory conditions and the reduction of IgG sialylation levels at least in female mice (86). However, indirect and direct B cell-intrinsic influences of nutrients or metabolites on B cell and PC signaling pathways regulating Ab glycosylation are still unclear and largely remain to be determined.

## Implications for inflammatory (autoimmune) conditions

As noted above, the effects of nutrient-sensing mechanisms on PC differentiation and secretion of class-switched pro-inflammatory (auto)Abs and cytokines might contribute to the induction of inflammatory (auto)immune mechanisms. Accordingly, inhibiting corresponding signaling pathways by nutrients and metabolites might inhibit B cell activation and pro-inflammatory (auto)immune PC responses or even shift B cell responses to anti-inflammatory PC responses. In the following we describe findings about the influences of nutrients and metabolites and signaling pathways on autoimmune responses.

In SLE, which can be considered a model disease for B cell-dependent inflammatory autoimmune disorders, histone H3 and H4 acetylation is reduced overall compared with healthy controls (89). Increasing histone acetylation *via* the pharmacological inhibition of class IIb HDAC6 decreased PC differentiation, Ab formation, and immune-complex mediated glomerulonephritis in murine SLE models (90, 91). Moreover, treatment with the HDAC inhibitors butyrate and valproic acid (synthetic SCFA) decreased the production of class-switched and hypermutated autoAbs by hyperacetylation of target host genes and subsequent upregulation of miRNAs in B cells, thereby ameliorating disease activity (55). Activation of the NAD^+^-dependent opposite acting HDAC, SIRT1, by the provision of resveratrol also resulted in decreased PC numbers, autoAb levels, and SLE-like disease.

In contrast, genetic deletion of the demethylases Tet2/Tet3 in murine B cells increased autoreactive B cell activity, production of autoAbs, and SLE-like disease by reduced recruitment of HDACs to the Cd86 locus (92).

The role of vitamin A and its derivatives in promoting PC differentiation and class-switch recombination has been extensively demonstrated. Accordingly, treatment of an SLE mouse model with vitamin A and retinoic acid significantly increased the B cell response and overall disease burden, although some protective effects on intestinal and renal pathology were registered as well (93). In contrast, some human case studies might indicate protective effects of vitamin A provision on SLE (94, 95) but require verification.

Since nutrient availability contributes to the activation and differentiation of B cells, caloric restriction is a potential therapeutic pathway. Indeed, caloric restriction inhibits the mTORC1 pathway in several immune cell types (96), reducing disease activity in models of MS (97, 98) and SLE (99) in congruence with the effects of pharmacological inhibition of mTORC1 (26, 100, 101). Regarding the B cell lineage, caloric restriction reduced the development of anti-nuclear antibody (ANA) depositions in genetically prone mice in comparison to the control chow diet and particularly a Western diet (86). Furthermore, high-fat and Western diets had a significant impact on the induction of total blood IgG Abs with low sialylation levels (82). B cells further mediate insulin resistance caused by high-fat diet feeding *via* the modulation of T cell function and the production of pathogenic autoAbs (102). Similarly, a high-fat diet induced a characteristic repertoire of Abs in visceral adipose tissue-resident B cells (103). Thus, ablation of B cells in high-fat diet-fed mice effectively abrogated the development of insulin resistance (102). These data suggest that high nutrient availability might support the development of inflammatory autoimmunity by increasing the production of autoreactive, class-switched Abs with changed glycosylation patterns by PCs. However, the metabolic pathways involved in developing autoreactive and particularly differentially glycosylated Abs remain elusive and warrant further experimental and clinical studies.

In addition to the production of Abs, B cells contribute to the immune response *via* the production of cytokines. In particular, the expression of anti-inflammatory cytokines by so-called regulatory B cells and PCs plays an important role in limiting inflammatory (auto)immune conditions (104–106). Notably, the SCFAs butyrate and pentanoate have been reported to be potent inducers of IL-10 in cells of the B cell lineage. Furthermore, butyrate inhibited class switching to the activating murine IgG subclasses (50). Since the levels of different SCFAs depend on the gut bacterial composition (107), these results suggest that the microbiota composition might affect PC differentiation and IL-10 induction *via* the production of different SCFAs making the gut bacterial composition an interesting target to manipulate inflammatory (auto)immune conditions.

Additionally, cholesterol synthesis and also vitamin A-signaling can contribute to IL-10 induction in B cells (86), indicating that a butyrate, pentanoate, and possibly vitamin A supply as well as a lack of external cholesterol – potentially inducing intracellular cholesterol synthesis – might protect against inflammatory reactions. Further studies are needed to discriminate which signals induce PC differentiation in general and which additional signals distinguish between the induction of pro- or anti-inflammatory phenotypes.

The experimental animal studies are further supported by real-life observations. Autoimmune diseases are much more prevalent in developed Western countries than in less industrialized regions (108). In addition to increased hygiene and decreased rates of infectious diseases, possible causes include dietary habits, summarized as “Western diets” (108, 109). Notably, Western diets are rich in sugars, salt, cholesterol, and saturated fat while lacking vitamins and dietary fibers (109). Thus, the high prevalence and increasing tendency of inflammatory autoimmunity in Western countries might be partially explained by an abundance of sugars, amino acids, and fats, while dietary precursors of vitamin A metabolites and SCFAs are missing (109). Thus, this dietary imbalance might cause immune dysregulation, including effects on B cell maturation toward PCs, as well as their functions. However, controlled clinical studies demonstrating PC-specific effects of dietary behavior are missing and are needed for validation.

In summary, preclinical studies indicate that nutrients and metabolites serve as crucial signaling molecules in the regulation of B cell activation and PC differentiation and function. Animal models of autoimmune diseases and epidemiologic associations indicate that effects on Ab-dependent and -independent functions of PCs might play a role in the pathogenesis of inflammatory autoimmune diseases. Metabolic signaling pathways that not only inhibit PC differentiation but also discriminate between pro- and anti-inflammatory PC functions are valuable targets that necessitate further evaluation in the future. Additional research is needed where evidence on PC-specific dietary effects in humans is missing or for additional nutrients that have generally been associated with positive effects on inflammatory autoimmune conditions, such as polyunsaturated fatty acids and phenolic acids. Dietary or pharmacological interventions aimed at the metabolic regulation of the B cell compartment are promising avenues for the prevention and treatment of inflammatory (auto)immune disorders and further preclinical and clinical studies could reveal the most efficient strategies.

## Author contributions

The conceptualization was done by BF and ME. The review results from the discussion and the consensus of all authors. The review was written by BF and ME. All authors contributed to the article and approved the submitted version.

## Funding

This study was supported by the Deutsche Forschungsgemeinschaft ((DFG, German Research Foundation) 429175970 (RTG 2633); 400912066 (EH 221/11-1); and 390884018 (Germany’s Excellence Strategies - EXC 2167, Precision Medicine in Chronic Inflammation (PMI)) (ME). JB was a PhD student of the RTG 2633. We acknowledge financial support by Land Schleswig-Holstein within the funding programme Open Access Publikationsfonds.

## Acknowledgments

Servier Medical Art (Les Laboratoires Servier, France, originals available at: https://smart.servier.com) under a Creative Commons Attribution 3.0 Unported License.

## Conflict of interest

The authors declare that the research was conducted in the absence of any commercial or financial relationships that could be construed as a potential conflict of interest.

## Publisher’s note

All claims expressed in this article are solely those of the authors and do not necessarily represent those of their affiliated organizations, or those of the publisher, the editors and the reviewers. Any product that may be evaluated in this article, or claim that may be made by its manufacturer, is not guaranteed or endorsed by the publisher.
